# Cross-cultural adaptation and psychometric properties of the Mainland Chinese version of the manchester orofacial pain disability scale (MOPDS) among college students

**DOI:** 10.1186/s12874-023-01976-8

**Published:** 2023-07-06

**Authors:** Yao Feng, Ze-Yue Ou-Yang, Jing-Jie Lu, Yi-Fan Yang, Qian Zhang, Meng-Mei Zhong, Ning-Xin Chen, Xiao-Lin Su, Jing Hu, Qin Ye, Jie Zhao, Ya-Qiong Zhao, Yun Chen, Li Tan, Qiong Liu, Yun-Zhi Feng, Yue Guo

**Affiliations:** 1 Department of Stomatology, The Second Xiangya Hospital, Central South University, Changsha, Hunan 410011 China; 2 Medical Psychological Center, the Second Xiangya Hospital of Central South University, Changsha, Hunan 410011 China

**Keywords:** Test translation and adaptation, Psychometric properties, Measurement invariance, Orofacial pain, Assessment

## Abstract

**Background:**

Orofacial pain (OFP) is a highly prevalent disorder in mainland China that predisposes to an associated physical and psychological disability. There is lack of a good properties mainland Chinese version of instrument to examine OFP. This study aims to cross-cultural adaptation and evaluate psychometrics properties of the Manchester Orofacial Pain Disability Scale (MOPDS) in mainland Chinese Mandarin context.

**Methods:**

Translation and cross-cultural adaption of the mainland Chinese version MOPDS were conducted following accepted guidelines of self-report measures. Chinese college students (N = 1039) completed the mainland Chinese version of the MOPDS for item analysis, reliability and validity tests, and measurement invariance analysis, and after a one-month interval, around 10% of the sample (n = 110) were invited to retest. To conduct the CFA and measurement invariance analysis, Mplus 8.4 was used. IBM SPSS Statistics 26 software were used for all additional studies.

**Results:**

We found that the mainland Chinese version of MOPDS contains 25 items, divided into two categories: physical disability and psychological disability. The scale demonstrated excellent internal reliability, test-retest reliability, and validity. The measurement invariance results proved that the scale could be applied to people of different gender, age, and health consultation status.

**Conclusions:**

The results demonstrated the mainland Chinese version of MOPDS has good psychometric properties and can be used to measure the level of physical and psychological disability of Chinese OFP peoples.

**Supplementary Information:**

The online version contains supplementary material available at 10.1186/s12874-023-01976-8.

## Background

In 2020, the Oral Pain Classification Committee proposed the first edition International Classification of Orofacial Pain (ICOP). An estimated 25% of adults report experiencing some form of orofacial pain(OFP), a broad term that includes a variety of painful conditions affecting the mouth, head, face, and neck regions [1, 2]. These conditions may involve different structures and originate from musculoskeletal, vascular, neurovascular, neurological, idiopathic, and psychogenic origins. Numerous studies have shown that OFP is associated with complex physical signs and symptoms and is often comorbid with other conditions such as depressive mood [3], which can cause severe physical and mental health burdens [4]. OFP can lead to functional limitations of the orofacial system such as painful mouth opening and painful occlusion, which can lead to physical disabilities such as interference with daily life and eating [5]. In addition, OFP can also cause anxiety or depression-like negative emotions, leading to an increase in the patient’s perception of pain [6]. Assessing the presence and consequences of pain and disability in orofacial is critical for quantifying physical and psychological impairment.

Using a scale, we can measure the impact of pain on physical and psychological disability, and people can self-assess through the scale, which is critical to their self-monitoring of pain. So far, there are many effective and reliable scales for assessing OFP-related symptoms, such as the Brief Pain Inventory–Facial scale (BPI-F) [7] and visual analogue scale (VAS) [8]. However, the most used scales primarily focus on physical disabilities. With the increasing attention given to OFPs associated with psychological disabilities, the ICOP proposed adding psychological scales to auxiliary diagnosis and treatment of patients. This evaluation method has also been applied by Staniszewski K et al. [9], resulting in a lengthy questionnaire in which the measurement results may not reflect psychological discomfort. Hence, applying the pain-related psychological disability scale would be a promising study. Aggarwal VR et al. published the Manchester Orofacial Pain Disability Scale (MOPDS) in 2005 [10]. In particular, the scale used a two-factor structure, including mental disability and physical disability, to assess orofacial and psychosocial limitations. Additionally, patients with low-flow vascular malformations [11] showed a higher psychological disability score when their pain worsened. In the general population [12], the scale has the advantages of brief content and convenient filling. The MOPDS is currently available in Brazilian-Portuguese, Chinese Cantonese, and Arabic [10, 12–14]. In particular, MOPDS has a broader range of applications than a single-dimensional scale such as VAS. In Monira Samaan Kallás et al. study, the VAS was used to assess the effectiveness of the Brazilian-MOPDS. Also, previous study proved that MOPDS could evaluate physical function and interference with daily life by chronic OFP [15, 16]. By completing the MOPDS, patients can facilitate personalized care management, screen for previously unidentified health problems, monitor disease prognosis and disease progression, make it easier for patients to communicate with health professionals and facilitate shared decision-making [15].

The official language of mainland China is Mandarin, Of the 900 million people in mainland China who speak Mandarin [17], many suffer from mental disorders caused by OFP [18]. Mandarin Chinese differs from Chinese Cantonese in some ways. For example, Cantonese has six tones, while Mandarin has four tones [19]. It is easy to cause comprehension problems for native mandarin-speaking people, so it is especially important to consider the use of Mandarin Chinese when studying mainland China groups, and it is unclear whether the results of the Cantonese study can be generalized to Mandarin speakers. Therefore, a mainland Chinese version of the self-screening scale is long overdue.

A study by Dorcas E. Beaton showed that to use the scale effectively in a new cultural context, it is necessary to go through Initial Translation, Synthesis of The Translations, Back Translation, Expert Committee, Test of the Prefinal Version, Submission of Documentation to the Developers or Coordinating Committee for Appraisal of the Adaptation Process these steps [20]. MOPDS’ Brazilian Portuguese was established through the above-described steps of intercultural adaptation. In addition, after cross-cultural adjustment, a questionnaire in another area should be tested for psychometric properties, such as construct validity, reliability, and internal consistency [21]. The Chinese version of mandibular function impairment questionnaire and the Chinese version of craniofacial pain and disability inventory both adopt the reliability and validity testing process after cross-cultural translation [22, 23]. After the scale’s psychometric properties have been validated, measurement invariance must be analyzed before it can be extensively tested, validated, and used in target populations. Furthermore, French and Finch found that the lack of measurement invariance can result in incorrect interpretation of differences between groups. So far, no measurement invariance has been done for the MOPDS [24].

After cross-cultural adaptation of the scale, appropriate target groups should be selected. According to the principles of the Translation and Cultural Adaptation Process for Patient-Reported Outcomes Measures (PROMS) [15], it is suggested the new translation usually with patients drawn from the target population to ensure that the purpose of including this step (e.g., ensuring that the translation is comprehensible to the general or patient population) could be met. A study of the association between professionals and the prevalence of OFP found students were considered high-risk groups [25]. In the original version of MOPDS [10], it was developed in 171 community subjects with self-reported OFP. Previous study has proved that university students had a high probability of suffering from OFP, which was one of self-related oral health and influenced the oral health-related quality of life of university students [26, 27]. In addition, the time of recruiting patients was during the Coronavirus disease (COVID-19) epidemic in China. It is difficult to recruit a large number of patients in offline outpatient clinics, but college students were broad and highly compliant, thus we selected a population of college students for our cross-cultural adaptation. Furthermore, some researchers found that transitioning from high school to a new social and academic environment is associated with a psychologically related disability such as depression [28]. If their oral health status is in a bad condition, it will adversely affect their mental health over time. Therefore, identifying underlying conditions and pain-related physical and psychological disabilities in college students can guide clinical diagnosis.

Firstly, this study assessed the prevalence of different types of OFP among college students, the regions where OFP was most frequently reported, the comorbidity of OFP, whose purpose was that Chinese college students as participants to assess the appropriateness and fidelity of the MOPDS scale. Then, this study to develop a mainland Chinese version of MOPDS, and to evaluate the tool’s use in Chinese college students’ demographics (sex, age, counseling status) in terms of reliability, validity, and measurement invariance. This survey has important research significance. Notably, the mainland Chinese version of MOPDS describes the group differences of Chinese college students and evaluates the clinical significance of OFP on mental and physical disabilities.

## Materials and methods

### Ethical considerations

This study was conducted in full accordance with the Code of Ethics of the World Medical Association (Declaration of Helsinki). The Human Experiment and Ethics Committee at the Second Xiangya Hospital of Central South University approved the study, which was carried out in accordance with the Declaration of Helsinki’s ethical principles (reference number: KQ2019FY01). Strengthening the Reporting of Observational Studies in Epidemiology guidelines were used as the foundation for reporting data [29]. The study was undertaken with the informed written consent of each participant. The privacy rights of the patients were always observed. A 2-step approach was used: (1) translation and cross-cultural adaptation, and (2) posterior validation among large Chinese college students. After signing an electronic informed consent form, eligible college students were invited to participate in an online questionnaire. Clinical questionnaire information was at the Open Science Foundation under https://osf.io/7dnvq/.

### The manchester orofacial pain disability scale (MOPDS)

Published in 2005, the MOPDS was the first self-administered scale for assessing OFP in community subjects and dental hospital patients [10]. MOPDS is composed of a primitive 32-item scale. Through rotated factor analysis, Aggarwal VR et al. recommend a 26-item scale, in which 19 items were assigned as principally psychosocial and 7 as principally physical [10]. The Cronbach’s alpha of MOPDS demonstrates good reliability (0.78 and 0.92 for the physical and psychosocial dimensions, respectively) and good internal consistency (values between 0.43 and 0.80). The answers to the scale are recorded on a 3 Likert scale: 0 = none of the time, 1 = on certain days, 2 = on most/every day, and covers 2 structures, namely, physical disability and psychosocial disability. This value is multiplied by the weight of each question to give a score range from 0 (best) to 52 (worst).

### Translation and cross-cultural adaptation process

After obtaining the authorization from the original author through E-mail, translation and cross-cultural adaption processes were conducted following accepted guidelines of self-report measures [20, 30]. The specific stages were as follows:

#### Stage I: initial translation

Two independent translations were completed by bilingual translators whose mother tongue is Chinese. Both translators have different backgrounds. One is an expert in Psychology (LJJ), and the other is an expert in stomatology (OYZY).

#### Stage II: synthesis of the translations

The study’s authors (FY and YYF) synthesized these translations (resulting in a general translation).

#### Stage III: back translation

Without knowing the original version, a non-native English teacher with no dental knowledge translated the scale into English. An important use of scales is to quantify the variable or indicator to be observed. Scales are developed to enable them to have the same test function across groups, contexts or control conditions, i.e. they have measurement invariance across groups, contexts and control conditions in the same language and culture [31]. Translation of the MOPDS is the process of converting the content of the scale from the original language (e.g. English) to the target language (e.g. Chinese). The method is a two-way translation (direct translation-back translation), as it maximizes semantic equivalence. The method model was proposed by Brislin [32] in 1970. The termination of translation is marked by the translator’s acceptance that the target language version accurately conveys the information of the original scale.

#### Stage IV: test of the prefinal version

The new scale was used to conduct field tests and interviews with 10 college students and 10 patients in the department of stomatology. Each subject completed the scale and was interviewed to understand what they thought each item meant. At this stage, these participants suggested that they ruled out the “sore to kiss” problem, proving that the item involved personal privacy and was difficult for people to answer positively.

#### Stage V: expert committee

A committee of experts reviewed the final version: the researchers included an expert in Pain (WYP), an expert in stomatology (FYZ), and two native English clinicians (O.A. & J.I.). A total of 10 experts were invited to evaluate the content validity based on the rule of the content validity index (CVI) [33]. According to the scale-level content validity index (S-CVI), 25 items were considered adequate, while the “sore to kiss” problem scored 0.5. The results of CVI are computed in Appendix-1.

#### Stage VI: synthesis of final documentation

The final stage was the submission of all the reports to the authors (FY and GY). As related to the item above, we decided to remove the question “sore to kiss,“ and the definitive 25-item version was finally obtained.

### Participants and data collection

Students at Hunan province participated in a cross-sectional scale study. During the first and second COVID-19 waves, taking into account social distance, the survey was conducted online. The questionnaires were designed on wenjuanxing and shared on social media such as wechat and QQ. We also distributed the questionnaires on social media channels in Hunan provinces and cities to increase the level of responsiveness. Eligible people completed the questionnaire by clicking on the link. A total of 1,039 college students completed an online scale and signed an electronic informed consent form during the duration of the study from October to December of 2021.

The first part of the scale includes students’ sociodemographic data and details of whether OFP is present and different characteristics of OFP in the past three months. In addition, students were asked to recall whether they had sought professional medical/dental consultation for OFP.

**Fig. 1:**
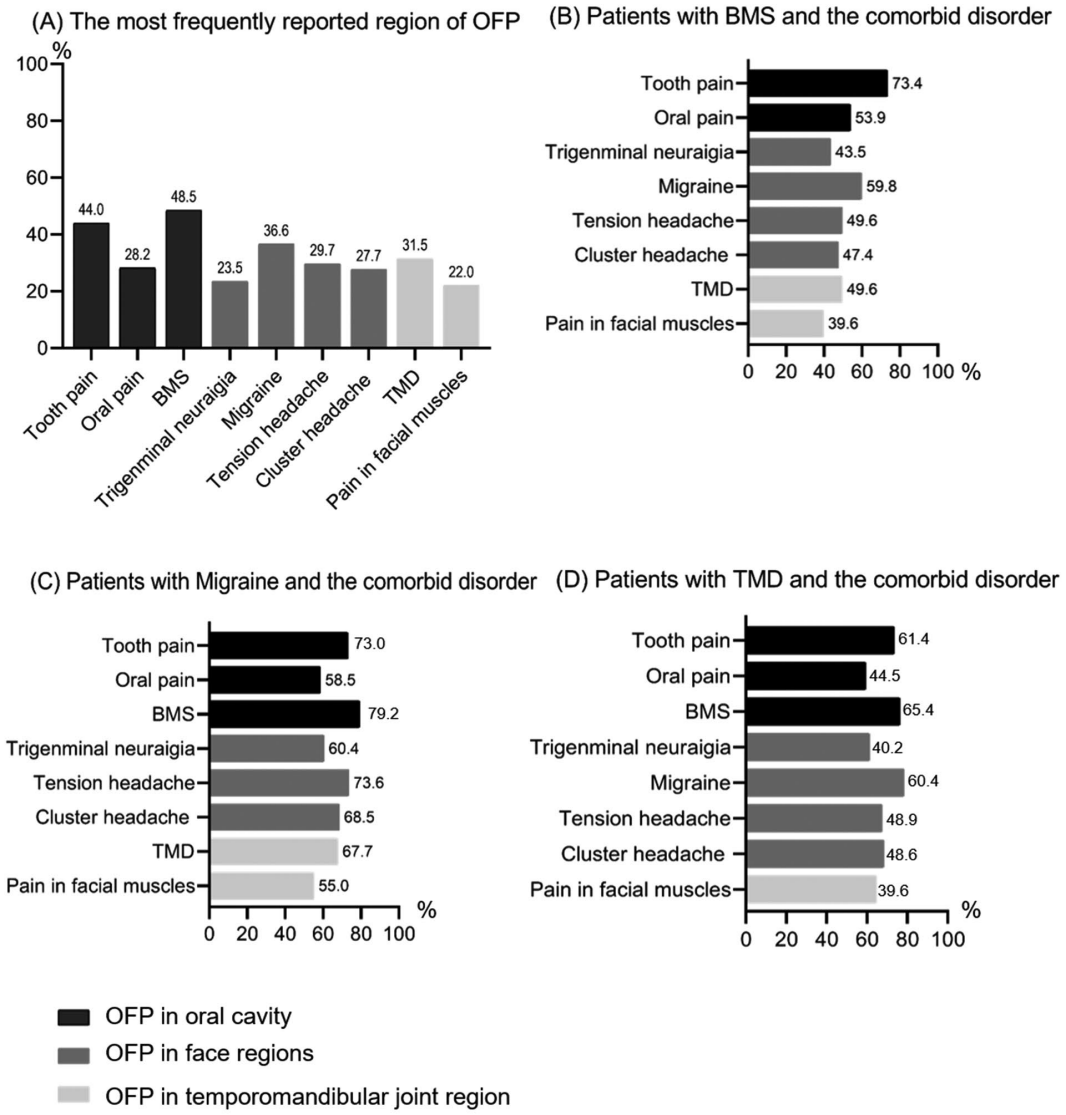
Prevalence of different types of OFP

OFP includes the following symptoms: OFP in the oral cavity:1) Tooth pain, 2) Oral (Mucous membrane, tongue) pain, 3) Burning sensation on mouth or tongue (burning mouth syndrome (BMS), Diagnostic criteria: Oral pain recurring daily for > 2 h per day for > 3 months; Pain has both of the following characteristics: (1) burning quality (2) felt superficially in the oral mucosa; Oral mucosa is of normal appearance, and local or systemic causes have been excluded; Not better accounted for by another ICOP or ICHD-3 diagnosis.) [2, 34]; OFP in face regions: 4) Trigeminal neuralgia, 5) Migraine, 6) Tension headache, 7) Cluster headache; OFP in Temporomandibular joint region: 8) Temporomandibular joint disorder (TMD), 9) Facial muscles pain, which based on ICOP [35], and the practice pointer of OFP recommend by Adonye Banigo et al. [36]. Sociodemographic characteristics of students with OFP are shown in Table 1. The prevalence of different types of OFP is shown in Fig. 1.

**Table 1:** Sociodemographic characteristics of university students

Presence of OFP	Yes(n = 682) (%)	No (n = 333) (%)	*χ * ^*2*^	*p*-value
Gender (n)				
Male (565)	321 (47.1)	244 (73.3)	62.2629	*p* < 0.001
Female (450)	361 (52.9)	89 (26.7)		
Age (n)				
16–19 years old (502)	291 (42.7)	211 (63.4)	38.3348	*p* < 0.001
20–30 years old (513)	391 (57.3)	122 (36.6)		
Educational (n)				
Bachelor (859)	565 (82.8)	294 (88.3)	7.4392	*p* = 0.024
Master (136)	105 (15.4)	31 (9.3)		
Doctor (20)	12 (1.8)	8 (2.4)		
Subject (n)				
Arts (287)	220 (32.3)	67 (20.1)	33.8444	*p* < 0.001
Science (328)	213 (31.2)	115 (34.5)		
Medicine (136)	103 (15.1)	33 (9.9)		
Others (264)	146 (21.4)	118 (35.4)		
Consulting history (n)				
Yes (290)	245 (35.9)	45 (13.5)	75.5884	*p* < 0.001
No (519)	287 (42.1)	232 (69.7)		
Missing (206)	150 (22.0)	56 (16.8)		

The second part of the survey is MOPDS, followed by the Chinese version of the short-form oral health impact profile-14 (OHIP-14) questionnaire. OHIP-14 scores were based on its seven dimensions: physiological discomfort, pain, physiologic inability, physical inability, function limitation, disability, and social inability. OHIP-14 scores from 0 to 4 based on a Likert scale: 0 - never, 1 - rarely, 2 - sometimes, 3 - frequently, and 4 - always. Summary scores were derived by summing the response across all 14 items, with lower OHIP scores indicating better oral health.

24 participants were excluded due to a lack of data in the questionnaire. For retest validation, around 10% of the sample (n = 110) were invited to retest the Chinese Mandarin version of MOPDS after a one-month interval between separate questionnaire administrations.

The sample size was determined to ensure the standards for using exploratory factor analysis (EFA), confirmatory factor analysis (CFA), and structural equation modeling (SEM) analysis and corresponded to the number of factors and observed variables of the proposed model. Based on Hair et al. (2014) [37], EFA consisting of seven factors and 30 manifest variables requires a sample size of 150 to 300. Based on Myers et al. (2011) [38], the minimum sample size required for CFA is 300. Furthermore, according to Hair et al. (2014) [38], SEM requires a sample size determined based on the number of factors and communalities value in EFA analysis. With the mentioned characteristics of the proposed model, the minimum sample size was 150 (with the value of communalities when analyzing EFA being from 0.5 or more) or 300 (with the value of communalities when analyzing EFA being from 0.45). Thus, this study used a sample size of 1016 to ensure that the conditions for performing the above analysis were satisfied.

### Statistical analysis

Confirmatory factor analysis (CFA) and measurement invariance analyses were performed with Mplus 8.3 with the robust weighted least squares with mean and variance adjustment estimator (WLSMV). All other analyses were conducted with the IBM SPSS version 26. Initially, sociodemographic characteristics were evaluated by the Chi-Squared Test.

#### Item analysis and reliability

Based on the variables in the factor analysis, we assigned each item to a scale and verified the scale’s internal consistency. Ordinal alpha estimated with polychromic correlations was used to assess the reliability criterion, and a value of alpha greater than 0.7 was considered acceptable [39]. Test-retest reliability refers to the concordance between the scores of repeated measurements from the same participant, which can be assessed by the intraclass correlation coefficient (ICC). A reliability coefficient of less than 0.4 is generally considered to indicate poor reliability and greater than 0.75 indicates good reliability [40].

#### Factor structure and validity

CFA can check the structural models of the MOPDS scale across the Chinese college students, that is, whether the MOPDS scale is good, whether the scale items are good, and whether the data collected reflects the expected results, is actually a validity test [41]. CFA allows for determining whether the factors of a scale are associated in the manner proposed by the researcher. In an operational sense, CFA was applied to model pre-defined constraints of the two-dimension structure of the Chinese Mandarin version of MOPDS and then to test how well the data fit the original model [10]. The output from CFA provided statistical information about the strength of relationships between individual items and the dimensions, and the correlation between dimensions. The average variance extracted (AVE) technique was used for demonstrating discriminant validity, and construct reliability (CR) was used for testing convergence validity.

The analysis data were categorical variables, and the weighted least squares mean and variance adjusted estimator was used to estimate the factor structure. Factor model fit was estimated using the χ² statistic, the comparative fit index (CFI), the Tucker–Lewis index (TLI), standardized root mean square residual (SRMR), and the root means the square error of approximation (RMSEA). Based on Hu and Bentler [42], CFI and TLI ≥ 0.95, SRMR < 0.05, and RMSEA ≤ 0.08 were considered to indicate a satisfactory fit. According to Fornell and Larker [43], discriminant validity is supported if the AVE for each construct is greater than its shared variance with any other dimensions. The convergence validity is good when CR is higher than 0.7 and the AVE is higher than 0.5. Henseler et al. (2015) [44] proposed the heterotrait-monotrait ratio (HTMT), and suggested that if the HTMT value is higher than 0.90 it indicates poor discriminant validity. Gold etal.(2001) [45]and Teo et al. (2008) [46]also used HTMT less than 0.9 as an evaluation criterion.

In addition, we compared the total score of the Chinese Mandarin version of MOPDS with the OHIP-14 to know the scale’s external validity. Floor and ceiling effects (i.e., number of respondents who achieved the lowest or highest possible scores) were examined. Floor or ceiling effects were considered a problem if more than 50% of a study population achieved the lowest or highest possible score [47].

#### Measurement invariance

To determine whether the measurement model could be equivalent across genders, age, and consulted status, multiple-group CFA was conducted to assess the measurement invariance of the Chinese Mandarin version of MOPDS [48].

The following invariance procedures were followed: (1) We tested the best-fit model from CFA across genders, age, and consulted status. (2) Configural invariance model (M1) was successfully estimated, and then constraints were sequentially added, including metric (weak) invariance model (M2), scalar (strong) invariance model (M3) and error variance invariance model (M4). A non-significant △χ² difference test (△χ² test) was considered as evidence of invariance. However, regarding the △χ² test sensitivity to sample size, we also relied on models’ differences in CFI (△CFI), where values < 0.010 △CFI [49, 50]. Changes in approximate fit indices remained low, supporting the weak, strong, strict, latent variance-covariance and latent mean invariance across genders, age, and consulted status. Then, the final grouped model was conducted in Table 4, based on the best fitting factor mode. T-test (T-test, *p* < 0.05) was applied to compare the statistically significant of each item between the different groups.

## Results

### Pilot testing

Twenty participants completed the pilot version of the Chinese Mandarin version of MOPDS: 10 college students and 10 patients from a dental clinic, based on translation and cultural adaptation principles [30]. In this case, they suggested deleting the “sore to kiss” problem, which was consistent with the decision of the committee of experts. the Chinese Mandarin version of MOPDS is shown in Appendix-4.

### Participants characteristics

1015 university students were prospectively included in the study, of which 565 (55.7%) were males and 450 (44.3%) were females (Table 1). There were 502 (49.5%) people aged between 16 and 20 years, while the rest were between 20 and 30 years. Of those who provided information on consultations (n = 809, 79.7%), 290 participants (35.8%) had consulted a healthcare professional for their pain, and 519 (64.2%) had not. The prevalence of OFP was significantly associated with gender (*p* < 0.001), age (*p* < 0.001), degree of education (*p* = 0.024), the field of study (*p* < 0.001) and consulting history (*p* < 0.001).

Participants were also asked about the specific history of OFP in an online survey. The most frequently reported region of OFP was oral cavity (n = 586, 57.7%), followed by face region (n = 423, 41.7%), and temporomandibular joint region (n = 336, 33.1%). Among them, BMS (n = 492, 48.5%) was the most frequently reported pain in the oral cavity, followed by the Migraine (n = 371, 36.6%) in the face region, and the TMD (n = 320, 31.5%) in the Temporomandibular joint region (Fig. 1(A)). OFP is characterized by substantial comorbidity. 73.4% patients with BMS comorbid tooth pain (Fig. 1(B)). In 79.2% of cases, BMS began after the comorbid Migraine (Fig. 1(C)), whereas BMS and migraine began after the comorbid TMD above 60% of cases (Fig. 1(D)). Moreover, most students had intermittent pain (91.8% in the oral cavity, 92% in the face region, 84.8% in the Temporomandibular joint region) while few students complained of continuous pain.

### Item analysis and reliability

To test the homogeneity of the scale, item-total correlations were used. All of the correlations above the recommended cut-off of 0.300 ranged from 0.573 to 0.745 [51]. Cronbach’s alpha was used to test the Chinese Mandarin version’s internal consistency. For the coefficient of the Chinese Mandarin version of MOPDS, the total score was 0.951. Cronbach’s alpha was 0.858 for physical disability and 0.946 for psychosocial disability. We also calculated the correlation of every item and how it would be affected by skipping 1 of them. After this, Cronbach’s alpha scores ranged from 0.948 to 0.951 for each item, reaching the optimal value (Appendix-2).

ICC was used to test the test-retest reliability. The results showed that the ICC was used to test the test-retest reliability. The results showed that the ICC was 0.737 [95%CI, 0.638-0.812] for the Chinese Mandarin version of MOPDS total (*p* < 0.001), 0.656 [95%CI, 0.535-0.751] for physical disability (*p* < 0.001), and 0.627 [95%CI, 0.498-0.728] for psychosocial disability (*p* < 0.001), suggesting an acceptable test-retest reliability.

### Factor structure and validity

Two-factor CFA model satisfied the recommended requirements (χ^2^(274) = 1122.413, *p* < 0.001; CFI = 0.976; TLI = 0.973; SRMR = 0.048; RMSEA (90%CI) = 0.055 (0.052–0.059). This factor structure fully replicated by findings from Monira et al. (factor loadings are reported on Table 2) [12].

**Table 2:** Confirmatory factor analysis: Item loadings, square roots of average variance extracted, and composite reliability for the MOPDS Chinese version

Item	Factor: 1 Physical disability	Factor 2 Psychosocial disability	AVE	CR
Item 1	0.846		0.748	0.947
Item 2	0.896			
Item 6	0.863			
Item 7	0.892			
Item 8	0.751			
Item 9	0.929			
Item 3		0.801	0.720	0.979
Item 4		0.897		
Item 5		0.784		
Item 10		0.858		
Item 11		0.910		
Item 12		0.736		
Item 13		0.844		
Item 14		0.898		
Item 15		0.811		
Item 16		0.897		
Item 17		0.852		
Item 18		0.833		
Item 19		0.814		
Item 20		0.874		
Item 21		0.890		
Item 22		0.852		
Item 23		0.873		
Item 24		0.851		
Item 25		0.822		

Note. AVE = average variance extracted; CR = construct relaibility

The correlation between psychosocial and physical dimensions was 0.751, therefore the shared variance = 0.751^2^ = 0.564001.The AVE for psychology and physiology were 0.748 and 0.720, respectively. Discriminant validity was established since the shared variance was smaller than the AVE for at least one of the constructs. By comparison, the CR of the CFA construct validity of the psychosocial dimension and physical dimension was above 0.700. The AVE values were above 0.500, whereby the correlation between the Chinese Mandarin version of MOPDS and the total score of OHIP was 0.622, indicating that the convergence validity was good. The Pearson correlation coefficient between the two factors was 0.751 and HTMT = 0.850. In this study, ceiling and floor effects were not identified. Within 15% of patients achieving the lowest or highest score in MOPDS, meeting the criteria, no ceiling/floor effect. A total of 0% (0/1015) of the participants had the lowest possible sum score and 30.5% (310/1015) the highest possible sum score on the MOPDS.

### Measurement invariance

For each group, the fit indices were computed (see Appendix-3). For the university students’ report groups (e.g., males and females), CFI was 0.958 or higher, TLI was 0.954 or higher, SRMR was 0.075 or lower, and RMSEA was 0.063 or lower.

The fit indices for the configural, metric, and scalar models of the Chinese Mandarin version of MOPDS are presented in Table 3. Metric (RMSEA = 0.052, SRMR = 0.057, and CFI = 0.981) and scalar (RMSEA = 0.049, SRMR = 0.057, and CFI = 0.982) invariance were supported across the gender of the university subjects. Regarding age, metric and scalar invariance (RMSEA = 0.051, SRMR = 0.056, and CFI = 0.976; RMSEA = 0.047, SRMR = 0.056, and CFI = 0.979) was supported. The metric (RMSEA = 0.049, SRMR = 0.051, and CFI = 0.986) and scalar (RMSEA = 0.046, SRMR = 0.051, and CFI = 0.987) invariance were found across consulted status. The results are showed in Table 3.

**Table 3:** Fit Indices for the Configural, Metric, and Scalar Models Across Gender, Age, Consulted Status

Group	Configural model			Metric model			Scalar model		
	RMSEA	SRMR	CFI	RMSEA	SRMR	CFI	RMSEA	SRMR	CFI
F vs. M	0.053	0.057	0.980	0.052	0.057	0.981	0.049	0.057	0.982
≥ 20 vs. ≤19	0.053	0.056	0.976	0.051	0.056	0.976	0.047	0.056	0.979
C vs. nC	0.050	0.051	0.986	0.049	0.051	0.986	0.046	0.051	0.987

Note. RMSEA = root mean square error of approximation; SRMR = standardized root mean square residual; CFI = comparative fit index; M = male; F = female; C = consulting history; nC = no consulting history

In the comparison of latent means (see Table 4), female students, university students 20 years or older reported higher total scores, particularly in the psychosocial disability dimension. Finally, students who consulted with a healthcare professional (compared with no consulting history) reported higher scores on the mainland Chinese version of MOPDS, especially in the dimension of physical disability.

**Table 4:** T-Difference Between the Latent Means of the Groups

The factors of MOPDS	F - M	≥ 20- ≤19 (age)	C - nC
Physical disability	1.335	5.450**	6.042**
Psychosocial disability	2.974*	7.272**	4.488**
Total scores	2.701*	7.177**	5.079**

Note. M = male; F = female; C = consulting history; nC = no consulting history

*p < 0.01. **p < 0.001

## Discussion

According to our study, Chinese college students are significantly affected by OFP. Additionally, MOPDS’s U.S. scale can also be translated into mainland Chinese culture and applied to Chinese university students. the mainland Chinese version of MOPDS were retained with 25 items based on international cross-cultural adjustment standards, and all respondents understood the contents. Psychiatric assessments demonstrated good reliability and validity of the mainland Chinese version of MOPDS. Gender, age, and consulted status measurement invariance were established, indicating the scale is an effective measurement tool.

Two hundred ninety study participants consulted a professional healthcare provider about OFP. This finding is more than the 25% reported by Setty S et al. [52]. We found that the prevalence of OFP was significantly associated with gender, age, degree of education, the field of study, and consulting history, and these findings are consistent with Aggarwal VR et al.‘s report [53]. The results in Fig. 1 showed that the most frequently reported region of OFP was the oral cavity. Of these, BMS was the most commonly reported oral pain, followed by primitive pain in the facial area and TMD in the temporomandibular joint area (Fig. 1A). Interestingly, a study by Sonja Smiljic et al. on a population of young adults in Serbia showed that regions with the highest pain prevalence were the temporal region and the region around the eye [54]. Therefore, this may reflect differences in pain sensitivity between races [55].

Interestingly, among Chinese college students, we found that OFP is characterized by many comorbidities. In 79.2% of cases, BMS started after concomitant migraine (Fig. 1(C)), whereas BMS and migraine started after concomitant TMD in more than 60% of cases (Fig. 1(D)). This is consistent with the findings of Gary D. Slade et al., who found cumulative effects of concurrent and previous episodes of TMD symptoms, headache, and body pain [56]. In addition, studies have shown that BMS, TMD, and migraine are strongly associated with psychological stress, especially with high psychological stress [57, 58]. Among college students, factors like stress are fairly common. Furthermore, the COVID-19 pandemic has had a significant impact on the lives and habits of university students, due to the necessity of social distance caused by the epidemic, and is a cause of difficulty and distress [59]. In addition, self-isolation affected the possibility for university students to fully experience university life, thus affecting academic learning. In the study by Odriozola-Gonzalez P, it was suggested that students had higher symptoms of anxiety and depression than the administrative and teaching staff of the university, indicating that students were the most psychologically affected by the COVID-19 [60]. In addition, 73.4% patients with BMS comorbid tooth pain (Fig. 1(B)), this may be because the site where BMS occurs is close to the mouth, and people tend to confuse it with toothache. By using a scale for evaluating physical and mental disabilities caused by OFP, college students can conduct a self-examination and get to the hospital if they need treatment.

Translation of MOPDS is based on the cross-cultural commissioning guidelines [30]. In the study of original version of MOPDS, 171community subjects with self-reported OFP and 48 dental hospital patients were recruited to test the construct validity of the instrument [26]. Then, a Portuguese language of MOPDS was developed, the authors excluded the questions “I am irritable, angry and easily frustrated”, and “I have lost earnings”. The Brazilian version of MOPDS shown high correlations compared to OHIP-14 and VAS [27].To ensure that the scale did not deviate from cultural norms and context during the translation process (especially the reverse translation stage), we validated the translated version to be more culturally aligned with mainland Chinese culture. Finally, the final version deleted the “sore to kiss” item because the CR level did not reach a significant level and retained 25 items.

The next step was to validate reliability in the university population since reliability is a prerequisite for scale consistency and stability. According the primary version of MOPDS [10], it is suggested that items 1, 2, 6, 7, 8, and 9 were related to physical disability, while the others (items 3, 4, 5, 10, 11, 12, 13, 14, 15, 16, 17, 18, 19, 20, 21, 22, 23, 24, 25) were related to psychosocial disability. All the mainland Chinese version of MOPDS items had a corrected item-total correlation exceeding recommended values, supporting their correlation with the overall scale. The reliability of the mainland Chinese version of the MOPDS is high, with a Cronbach’s alpha of 0.951 for the total scale score and a score between 0.858 (physical disability) and 0.946 (physical disability) for the two dimensions. The original scale, the Brazilian-Portuguese version [12], the Arabic version [13], and the Cantonese version [14] all also verified these two dimensions, indicating good internal consistency. Previous studies showed that MOPDS is reasonable to assume that people who consulted a healthcare professional because of orofacial pain would report greater score of disability than those who did not [10]. Because the questionnaire can effectively provide a useful means for patients to describe their pain more clearly by indicating the levels of associated disability, MOPDS have been widely applied in clinical measures, such as low-flow vascular malformations [11], masticatory myofascial pain [61], chronic OFP [16]. Thus, this study would prove useful in epidemiological studies examining the etiology of facial pain syndromes. In addition, the ICC for the total scale was 0.737, the ICC for physical disability was 0.656 (*p* < 0.001), and the ICC for psychosocial disability was 0.627 (*p* < 0.001), demonstrating excellent test-retest reliability.

For the factor structure and validity of the scale, we conduct CFA to identify potential domains of the mainland Chinese version of MOPDS. Twenty-five items of the measure were divided into two factors, and factor loadings of all items were > 0.400. The original and the mainland Chinese version of MOPDS determined a two-factor structure formed by the “psychosocial dimension” and “physical dimension” [10]. These results demonstrated that the measure had good structural validity. Furthermore, the correlation between the scores of Brazil-MOPDS and OHIP-14 was high, r = 0.857 [12]. Therefore, the scale can detect the mental disability and physical disability dimensions of OFP in Chinese culture.

Based on the results on reliability and validity, we performed measurement invariance. the mainland Chinese version of MOPDS’s configural, metric, scalar, and error invariance are all acceptable and had measurement invariance across age, consult status, and gender. This is the first study that has measured invariance in the MOPDS scale. The mainland Chinese version of MOPDS total scores for female students in Table 4 was higher than those for male students, especially in the psychosocial disability dimension, suggesting that women might be more sensitive to pain than men [62]. A significant difference exists between men and women in how they perceive and manage pain, which may impact therapeutic interventions [63]. In college students 20 and older, total mainland Chinese MOPDS scores were higher, especially in psychological areas. In certain studies, there is a greater likelihood of anxiety symptoms in sophomores, juniors, and seniors due to differences in curriculum design [64]. Students who consulted with a healthcare professional reported higher mainland Chinese MOPDS scores, especially regarding physical disability, suggesting that healthcare professional consultations may influence disability risk [65]. Clinical practitioners can address adherence through counseling and accountability for individual patients [53]. Therefore, our results suggest that the mainland Chinese version of MOPDS can be effective in assessing the college student population.

For patients with OFP, it is more challenging to perceive and only becomes obvious when there is related swelling. The severity of a patient’s facial pain is frequently underrated, even by medical professionals. By indicating the degrees of associated physical and psychological disability, our study of the Chinese version of MOPDS could help patients more effectively describe their pain. Additionally, by demonstrating measurement invariance, the comparative study of gender, age, and counseling status among Chinese college students is efficient and understandable. According to these findings, Chinese university students could conduct research using the MOPDS version from the mainland China. It can be developed further to help doctors and patients self-diagnose in the future. This ought to be helpful in epidemiological investigations looking at the causes of OFP syndromes. Additionally, resources can be concentrated on conditions that are more incapacitating by determining the public health burden of particular orofacial pain conditions.

The study had some notable limitations regarding the size of the study. This was a cross-sectional study; therefore, our study suffered from the typical limitations of a cross-sectional analysis. Therefore, additional research should expand the study’s scope and the number of participants to determine added reliability and efficacy of the scale.

## Conclusions

The mainland Chinese version of MOPDS provides an objective tool for assessing pain and disability in Chinese college students that is well structured, internally consistent, reproducible and validated. By establishing measurement invariance, the comparative study of Chinese college students’ gender, age, and consulted status are effective and interpretable. This is the tool for self-assessment of OFP in mainland China, which can be further expanded in the future to assist clinicians and patients in self-diagnosis. Chinese university students presented differences in profiles, which may support the concept of individualized pain mechanisms-based management.

## Electronic supplementary material

Below is the link to the electronic supplementary material.

Supplementary Material 1

## Data Availability

The datasets generated during the current study are available in the Open Science Framework (OSF) repository: https://osf.io/7dnvq/.

## Abbreviations

ICOP: International Oral Pain Classification
OFP: Orofacial pain
VAS: Visual analogue scale
MOPDS: The Manchester Orofacial Pain Disability Scale
COVID: Coronavirus disease
TMD: Temporomandibular joint disorder
CFA: Confirmatory factor analysis
ICC: Intraclass correlation coefficient
AVE: Average variance extracted
CR: Construct reliability
CFI: Comparative fit index
TLI: Tucker–Lewis index
SRMR: Standardized root mean square residual
RMSEA: Root means the square error of approximation
BMS: Burning mouth syndrome
PROMS: Patient-Reported Outcomes Measures
OHIP-14: Oral health impact profile-14
EFA: Exploratory factor analysis
CFA: Confirmatory factor analysis
SEM: Structural Equation Model
HTMT: Heterotrait-monotrait ratio
WLSMV: Weighted least squares with mean and variance adjusted
