# Supplementary material for: Cross-cultural adaptation and psychometric properties of the Mainland Chinese version of the manchester orofacial pain disability scale (MOPDS) among college students
Source: BMC Med Res Methodol. 2023 Jul 6;23:159. doi: 10.1186/s12874-023-01976-8 (PMC10324202; doi:10.1186/s12874-023-01976-8)
Supplement: Supplementary file 1 — Supplementary Material 1 [file 12874_2023_1976_MOESM1_ESM.docx]

Appendix-1: Evaluation of I-CVI with 10 assessments of expert agreements

| Item | Experts rating | | | | | | | | | | Number Giving Rating of 3 or 4 | I-CVI | Pc | K* | Evaluation |
| --- | --- | --- | --- | --- | --- | --- | --- | --- | --- | --- | --- | --- | --- | --- | --- |
|  | 1 | 2 | 3 | 4 | 5 | 6 | 7 | 8 | 9 | 10 |  |  |  |  |  |
| 1 | 3 | 4 | 3 | 3 | 3 | 4 | 4 | 4 | 4 | 4 | 10 | 1 | 0.001 | 1.00 | Excellent |
| 2 | 2 | 4 | 4 | 3 | 3 | 3 | 3 | 4 | 4 | 4 | 9 | 0.9 | 0.010 | 0.90 | Excellent |
| 3 | 3 | 3 | 4 | 4 | 4 | 4 | 4 | 3 | 4 | 4 | 9 | 0.9 | 0.010 | 0.90 | Excellent |
| 4 | 2 | 2 | 4 | 3 | 4 | 3 | 3 | 3 | 4 | 4 | 8 | 0.8 | 0.044 | 0.79 | Excellent |
| 5 | 4 | 4 | 4 | 4 | 4 | 4 | 4 | 4 | 4 | 4 | 10 | 1 | 0.001 | 1.00 | Excellent |
| 6 | 1 | 1 | 2 | 2 | 3 | 3 | 2 | 3 | 3 | 3 | 5 | 0.5 | 0.246 | 0.34 | Fair |
| 7 | 4 | 4 | 4 | 4 | 4 | 4 | 4 | 4 | 3 | 4 | 10 | 1 | 0.001 | 1.00 | Excellent |
| 8 | 4 | 4 | 3 | 3 | 2 | 3 | 4 | 4 | 4 | 4 | 9 | 0.9 | 0.010 | 0.90 | Excellent |
| 9 | 4 | 3 | 4 | 3 | 3 | 4 | 4 | 4 | 4 | 4 | 10 | 1 | 0.001 | 1.00 | Excellent |
| 10 | 4 | 4 | 4 | 4 | 4 | 4 | 4 | 3 | 4 | 4 | 10 | 1 | 0.001 | 1.00 | Excellent |
| 11 | 4 | 4 | 4 | 4 | 4 | 4 | 3 | 3 | 4 | 4 | 10 | 1 | 0.001 | 1.00 | Excellent |
| 12 | 3 | 2 | 2 | 4 | 3 | 3 | 3 | 3 | 3 | 4 | 8 | 0.8 | 0.044 | 0.79 | Excellent |
| 13 | 3 | 2 | 2 | 4 | 3 | 3 | 3 | 3 | 3 | 3 | 8 | 0.8 | 0.044 | 0.79 | Excellent |
| 14 | 3 | 3 | 3 | 3 | 4 | 4 | 4 | 4 | 4 | 4 | 10 | 1 | 0.001 | 1.00 | Excellent |
| 15 | 4 | 4 | 3 | 3 | 4 | 3 | 4 | 3 | 3 | 3 | 9 | 0.9 | 0.010 | 0.90 | Excellent |
| 16 | 3 | 3 | 3 | 3 | 3 | 3 | 3 | 2 | 2 | 3 | 8 | 0.8 | 0.044 | 0.79 | Excellent |
| 17 | 4 | 4 | 4 | 4 | 4 | 3 | 4 | 3 | 4 | 3 | 10 | 1 | 0.001 | 1.00 | Excellent |
| 18 | 2 | 3 | 3 | 3 | 3 | 3 | 3 | 4 | 4 | 4 | 9 | 0.9 | 0.010 | 0.90 | Excellent |
| 19 | 4 | 4 | 3 | 3 | 3 | 3 | 3 | 3 | 3 | 3 | 10 | 1 | 0.001 | 1.00 | Excellent |
| 20 | 3 | 3 | 3 | 3 | 3 | 4 | 4 | 4 | 4 | 4 | 10 | 1 | 0.001 | 1.00 | Excellent |
| 21 | 1 | 2 | 3 | 3 | 3 | 3 | 3 | 4 | 3 | 4 | 8 | 0.8 | 0.044 | 0.79 | Excellent |
| 22 | 3 | 3 | 2 | 3 | 3 | 3 | 3 | 3 | 4 | 4 | 9 | 0.9 | 0.010 | 0.90 | Excellent |
| 23 | 4 | 4 | 4 | 3 | 4 | 3 | 3 | 4 | 4 | 4 | 10 | 1 | 0.001 | 1.00 | Excellent |
| 24 | 3 | 3 | 3 | 4 | 4 | 4 | 4 | 4 | 4 | 3 | 10 | 1 | 0.001 | 1.00 | Excellent |
| 25 | 3 | 4 | 4 | 3 | 4 | 4 | 4 | 3 | 3 | 3 | 10 | 1 | 0.001 | 1.00 | Excellent |
| 26 | 3 | 2 | 2 | 3 | 4 | 4 | 2 | 4 | 3 | 4 | 8 | 0.8 | 0.044 | 0.79 | Excellent |

Appendix-2: Descriptive statistics and internal consistency of the Chinese Mandarin version of MOPDS.

| Item | Mean | SD | Corrected item total correlation | If item dropped, Cronbach’s alpha |
| --- | --- | --- | --- | --- |
| Item 1 | 0.19 | 0.436 | 0.616 | 0.949 |
| Item 2 | 0.11 | 0.355 | 0.624 | 0.949 |
| Item 3 | 0.22 | 0.466 | 0.635 | 0.949 |
| Item 4 | 0.11 | 0.35 | 0.677 | 0.949 |
| Item 5 | 0.21 | 0.466 | 0.607 | 0.949 |
| Item 6 | 0.19 | 0.447 | 0.625 | 0.949 |
| Item 7 | 0.17 | 0.414 | 0.660 | 0.949 |
| Item 8 | 0.22 | 0.447 | 0.531 | 0.950 |
| Item 9 | 0.13 | 0.389 | 0.671 | 0.949 |
| Item 10 | 0.14 | 0.386 | 0.682 | 0.949 |
| Item 11 | 0.10 | 0.349 | 0.680 | 0.949 |
| Item 12 | 0.19 | 0.519 | 0.497 | 0.951 |
| Item 13 | 0.24 | 0.477 | 0.694 | 0.948 |
| Item 14 | 0.12 | 0.367 | 0.706 | 0.948 |
| Item 15 | 0.31 | 0.560 | 0.632 | 0.949 |
| Item 16 | 0.15 | 0.409 | 0.733 | 0.948 |
| Item 17 | 0.16 | 0.421 | 0.679 | 0.948 |
| Item 18 | 0.15 | 0.420 | 0.631 | 0.949 |
| Item 19 | 0.35 | 0.567 | 0.645 | 0.949 |
| Item 20 | 0.33 | 0.523 | 0.687 | 0.948 |
| Item 21 | 0.12 | 0.358 | 0.697 | 0.948 |
| Item 22 | 0.16 | 0.407 | 0.681 | 0.948 |
| Item 23 | 0.22 | 0.464 | 0.715 | 0.948 |
| Item 24 | 0.27 | 0.493 | 0.670 | 0.949 |
| Item 25 | 0.20 | 0.443 | 0.637 | 0.949 |

Appendix-3: Confirmatory factor analysis for which groups measurement invariance was examined across.

| Group (n) | CFI | TLI | SRMR | RMSEA |
| --- | --- | --- | --- | --- |
| Male (565) | 0.990 | 0.989 | 0.037 | 0.042 |
| Female (450) | 0.958 | 0.954 | 0.075 | 0.063 |
| 16-19 years old (502) | 0.978 | 0.976 | 0.057 | 0.048 |
| 20 and above (513) | 0.973 | 0.971 | 0.055 | 0.058 |
| Have a health consultation (290) | 0.987 | 0.986 | 0.052 | 0.054 |
| No health consultation (519) | 0.984 | 0.982 | 0.050 | 0.050 |

Appendix-4: The Chinese Mandarin version of MOPDS questionnaire

Below are some statements about associated issues people have because of pain in their face, mouth, or jaws. Please recall whether you have had any related pain problems in the past 3 months， and were these problems only on some days or on most or every day in the past 3 months?

|  | Because of pain in their face, mouth or jaws. | During the past month, this has applied to me: (please tick On line  under appropriate statement) | | |
| --- | --- | --- | --- | --- |
|  |  | None of the time | On some days | On most days/every day |
| 1 | I cannot open my mouth as wide as I could |  |  |  |
| 2 | I cannot touch my face |  |  |  |
| 3 | I have difficulty falling asleep |  |  |  |
| 4 | I wake up at night in pain |  |  |  |
| 5 | I cannot find a comfortable position in which to sleep |  |  |  |
| 6 | I cannot eat hard foods |  |  |  |
| 7 | I take longer to finish my meals |  |  |  |
| 8 | I no longer enjoy my food |  |  |  |
| 9 | I find it difficult to smile or laugh |  |  |  |
| 10 | People find me difficult to live with |  |  |  |
| 11 | I have had to take time off work |  |  |  |
| 12a | I have lost earnings |  |  |  |
| 13 | I have found it difficult to concentrate |  |  |  |
| 14 | I have problems performing normal household tasks |  |  |  |
| 15 | I would rather be by myself |  |  |  |
| 16 | I find it difficult to talk for long periods of time |  |  |  |
| 17 | I have canceled social activities and holidays |  |  |  |
| 18 | I am unable to eat out in restaurants |  |  |  |
| 19 | I feel weary/tired |  |  |  |
| 20 | I am irritable, angry and easily frustrated |  |  |  |
| 21 | I cannot stop crying |  |  |  |
| 22 | I am worried that I may have a serious illness |  |  |  |
| 23 | I feel embarrassed and self-conscious |  |  |  |
| 24 | I feel depressed |  |  |  |
| 25 | I feel I no longer take any pleasure in life |  |  |  |
